# Chronicity in the 21^st^ century: facing the challenges of a changing society

**DOI:** 10.1590/0034-7167.2023760401

**Published:** 2023-09-04

**Authors:** Namie Okino Sawada, Silvana Maria Coelho Leite Fava, Bianca de Moura Peloso-Carvalho

**Affiliations:** I Universidade Federal de Alfenas. Alfenas, Minas Gerais, Brasil

Currently, there is an increase in non-communicable diseases (NCDs), population aging and changes in lifestyle, which corroborates the increase in chronicity, one of the greatest challenges faced by contemporary society. In this editorial, we will examine chronicity in the 21^st^ century and the need to address this problem in a comprehensive and sustainable way.

In recent years, we have witnessed a significant increase in the number of people with chronic diseases such as diabetes, cardiovascular diseases, cancer, chronic respiratory diseases, among others. These diseases have a profound impact on people’s quality of life, because they impose adaptations on the way of life and a substantial economic burden on families and health systems, which need to be understood in the care management process in the Health Care Network.

A Brazilian study showed that, in the last decade, there was an increase in the prevalence of depression, cancer, diabetes, neuropsychiatric disorders, chronic lung problems and musculoskeletal disorders. Those Brazilians affected with at least one NCD, over time, were covered by the Family Health Strategy, but there was a reduction in timely medical care and obtaining free prescription drugs^(1)^. Thus, it is crucial to understand the underlying causes to support effective public policies for NCD prevention and control.

Another important factor that contributes to chronicity is the aging of the population. Technological advances and better living conditions have led to longevity; however, aging leads to an increase in the prevalence of chronic diseases, which requires an adaptation of the health system to meet older adults’ demands, providing adequate, comprehensive and quality care.

The globalized and urbanized world brought changes in lifestyle that impacted the emergence of chronic diseases. The majority of the population has adopted unhealthy habits, such as fatty diets, sedentary lifestyle, alcoholism and smoking, which have contributed to the increase of these diseases. Thus, programs to promote healthy lifestyles and awareness of associated risks are essential to prevent and control chronicity, in which the health system must promote primary, secondary, tertiary and quaternary prevention based on evidence.

Chronicity constitutes a major challenge for the health system, since resources are limited, health services are not integrated and care is often fragmented. To overcome these challenges, an interprofessional and collaborative approach is needed, involving health professionals, governments, academic institutions and civil society, with the aim of developing innovative and sustainable approaches in managing this situation.

In view of this, in order to achieve an integrated approach to chronic health care, it is necessary to transition from a model focused on the disease to a model centered on integrated and person-centered care^(2)^, being increasingly encouraged by international guidelines, with an emphasis on health promotion for disease prevention with self-management and effective coordination of care. Using technologies, such as telemedicine and artificial intelligence, can help in the monitoring, diagnosis of diseases and follow-up of people with NCDs. A systematic review on machine learning has shown that the machine can learn to predict the occurrence of individual chronic diseases and disease progression, and their determinants, in many contexts. These findings are original and relevant to improving clinical decisions and the organization of health services^(3)^.

It is also worth mentioning the United Nations (UN) 2030 agenda, which advocates Sustainable Development with principles of action by humanity and its different forms of society, to ensure their survival on the planet in conditions of equity and social justice for all and for future generations^(4)^. In goal 7, it is established:

[...] a world free of poverty, hunger, disease and want, where all life can thrive. We envisage a world free of fear and violence. A world with universal literacy. A world with equitable and universal access to quality education at all levels, to health care and social protection, where physical, mental and social well-being are assured. A world where we reaffirm our commitments regarding the human right to safe drinking water and sanitation and where there is improved hygiene; and where food is sufficient, safe, affordable and nutritious. A world where human habitats are safe, resilient and sustainable and where there is universal access to affordable, reliable and sustainable energy^(4)^.

In these actions, chronicity stands out, which requires decisive and collaborative action with comprehensive health policies, focused on prevention, health education and integrated care, aiming at sustainable development, ensuring the quality of life of the population and helping to build a healthier, more resilient and productive society.
